# Satellite Observations of NO_**2**_ Trend over Romania

**DOI:** 10.1155/2013/261634

**Published:** 2013-12-25

**Authors:** Daniel-Eduard Constantin, Mirela Voiculescu, Lucian Georgescu

**Affiliations:** European Center of Excellence for the Environment, Faculty of Sciences and Environment, Dunarea de Jos University of Galati, Domneasca Street, No. 111, 800201 Galati, Romania

## Abstract

Satellite-based measurements of atmospheric trace gases loading give a realistic image of atmospheric pollution at global, regional, and urban level. The aim of this paper is to investigate the trend of atmospheric NO_2_ content over Romania for the period 1996–2010 for several regions which are generally characterized by different pollutant loadings, resulting from GOME-1, SCIAMACHY, OMI, and GOME-2 instruments. Satellite results are then compared with ground-based in situ measurements made in industrial and relatively clean areas of one major city in Romania. This twofold approach will help in estimating whether the trend of NO_2_ obtained by means of data satellite retrievals can be connected with the evolution of national industry and transportation.

## 1. Introduction

Nitrogen dioxide (NO_2_) is a trace gas with important impact on atmospheric chemistry and human health. NO_2_ is created by both natural and anthropogenic (human) processes. Natural sources of NO_2_ are lightning and microbial activity in soil by the oxidation of ammonium nitrate while the main anthropogenic sources of NO_2_ are surface transport, power generation, industrial processes, air transport, and biomass burning [1]. Tropospheric NO_2_ has a relatively short lifetime of the order of hours to days, depending on season, latitude, and altitude [2, 3]. However, high concentrations can be found close to emission sources.

The first global results about NO_2_ observed from space were presented after the launching of GOME-1 (Global Ozone Monitoring Experiment) instrument aboard ERS-2 satellite [4]. These were followed by results from other satellite instruments: SCIAMACHY (SCanning Imaging Absorption spectroMeter for Atmospheric CHartographY) in 2002 aboard Envisat [5], OMI (Ozone Monitoring Instrument) in 2004 aboard EOS-Aura [6], and GOME-2 aboard MetOp [7]. The retrieval method is based on the measurements of solar radiation reflected by the atmosphere and surface of Earth in nadir view (GOME-1, OMI, and GOME-2) or limb-nadir view (SCIAMACHY). Some characteristics of each instrument are presented in Table 1.

Recent studies based on measurements from space show that daily, weekly, or seasonal NO_2_ variations in various industrialized parts of world, which hereafter will be called “hot spots,” can be detected with satellite-borne instruments. Due to the increasing horizontal resolution of satellite instruments, information about the magnitude and life-time of various hot spots can be acquired [8]. Space-borne measurements offer also information about long-range transports of pollutants through the atmosphere [9]. Satellite observations can be used for detecting daily or weekly variability at urban level. Ordóñez et al. in [10] compared GOME-1 measurements with in situ ground-based measurements converted to boundary layer columns around Milan, Italy, and found a very good agreement for relatively low polluted areas. Zyrichidou et al. in [11] compared results over 32 locations from the South-Eastern Europe and concluded that discrepancies exist between various satellite measurements due to the different local overpass times of each satellite, horizontal resolution, and local NO_2_ diurnal variability.

Beirle et al. [2] and Boersma et al. [12] observed a significant reduction of tropospheric NO_2_ on Sundays for Europe, USA, and Japan, Using GOME-1, respectively SCIAMACHY and OMI measurements, Beirle et al. [2] and Boersma et al. [12] observed a significant reduction of tropospheric NO_2 _on Sundays for Europe, USA and Japan. Decreases of tropospheric NO_2_ columns on free days (according to various societal regulations or religious beliefs, i.e., Saturdays in Israel and Fridays in Islamic countries) were observed also in the Middle East. No weekly cycle was observed in China, for instance, due to the superimposition of different economic, cultural, and religious backgrounds [12]. In the same paper [12], the seasonal variation observed from SCIAMACHY and OMI sensors using in situ surface measurements in Israeli cities was analyzed. The NO_2_ surface concentration was converted in boundary layer columns and it was found that some differences between satellite data exist; the NO_2_ content estimated by SCIAMACHY was lower than that by OMI measurements with up to 40% during the summer.

The trend of tropospheric NO_2_ at continental and regional level using the satellite measurements was analyzed in [13] using GOME and SCIAMACHY data for 1996–2006. A significant reduction in NO_2_ was observed for Europe and Eastern United States while China, Iran, and Russia are characterized by strong increases [13–15]. In [16], the effects of economic downturn from China for 2008-2009, visible in tropospheric concentrations of NO_2_ detected from space, are presented. Emissions from international shipping detected from space were presented in [17], based on SCIAMACHY data from the navigable sector of the Red Sea.

In this paper, the NO_2_ trend for the period 1996–2010 over Romania is analyzed, using available satellite measurements by GOME-1, SCIAMACHY, OMI, and GOME-2. Different satellite data are compared for seven locations with various levels of pollution: high, medium, and low. Particularities of each location are considered, together with seasonal characteristics, in order to evaluate the causes and consequences of differences between the satellite data. In the second part of the paper, a detailed analysis is presented for the capital of Romania, Bucharest, which is the largest city of Romania. Ground-based data are presented and compared with results of satellite observations.

## 2. Methodology and Data

**Figure 1:**
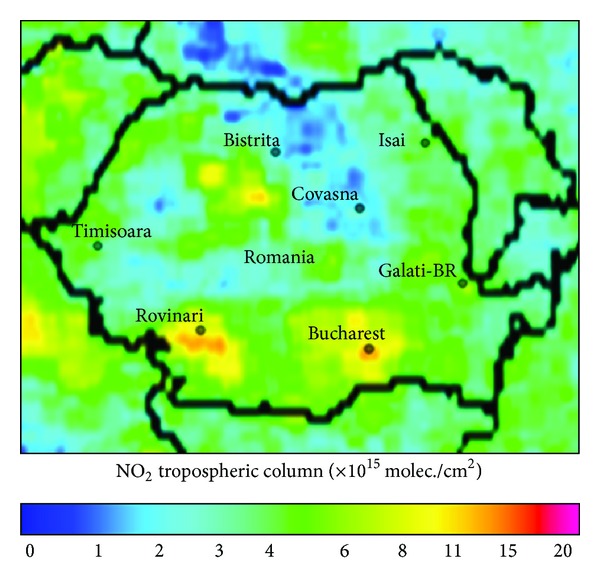
Spatial distribution of the selected cities using a NO_2_ OMI map (December 2005).

The seven representative cities of Romania selected for the present analysis are Bucharest, Rovinari, Timisoara, Iasi, Galati-Braila (considered as one urban agglomeration, since they are very closely located, practically inside one satellite pixel), Covasna, and Bistrita and are listed in Table 2. They have different NO_2_ pollution levels (low, medium, and high) and are located in different regions, thus covering a large part of the country (Figure 1). The NO_2_ loading in Romania, with a tropospheric monthly mean VCD (vertical column density) of about 11 × 10^15^ molecules/cm^2^ (as given by OMI for Bucharest), is two times smaller than in other polluted areas such as the central and eastern parts of China, Eastern Coast of USA, or Western Europe, as shown in [14, 16].

Monthly means of tropospheric NO_2_ VCD derived from four satellite instruments (GOME-1, SCIAMACHY, OMI, and GOME-2), collected between 1996 and 2010 (Table 1), were used for the present study. These are based on slant column NO_2_ retrievals with the DOAS technique [18]. Slant columns from GOME, SCIAMACHY, and GOME-2 observations are derived by BIRA-IASB, the slant columns from OMI by KNMI/NASA. The description of retrieval of the tropospheric column can be found in [19–21]. For this study, we use tropospheric NO_2_ columns available from the Tropospheric Emission Monitoring Internet Service (http://www.temis.nl) version 1 for satellite instruments.

**Figure 2:**
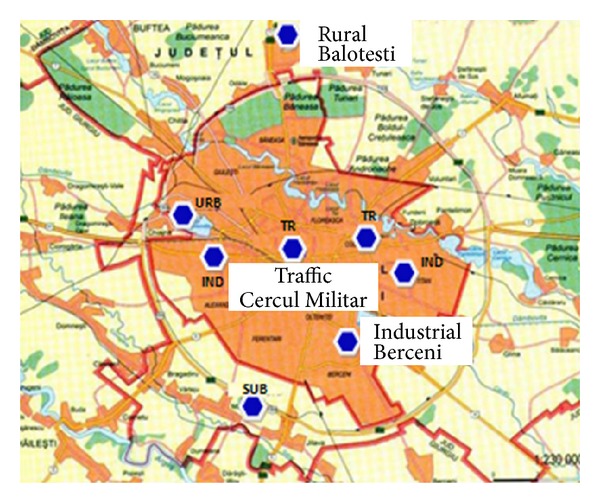
Locations of in situ monitoring stations in Bucharest.

The investigation was limited to the city areas based on the approximate geometrical centre of the city, in tetragons of 0.25°. To remove the possible effects of different spatial resolutions, loadings given by OMI, with a smaller spatial resolution (0.125°), were converted into larger grids to meet GOME-1, SCIAMACHY, and GOME-2 horizontal resolution. In the second part of the paper, which focuses on a detailed analysis made for a major city (Bucharest), in situ measurements of NO_2_ at ground level were compared to satellite measurements. NO_2_ concentrations in Bucharest city are recorded by eight monitoring stations equipped with chemiluminescence detectors. The locations of the monitoring stations are represented in Figure 2 and are grouped in traffic monitoring, industrial monitoring, and urban, suburban, and rural monitoring. The in situ NO_2_ measurements used in this work represent the monthly average of the daily observations from the monitoring stations presented in Figure 2.

## 3. Results 

**Figure 3:**
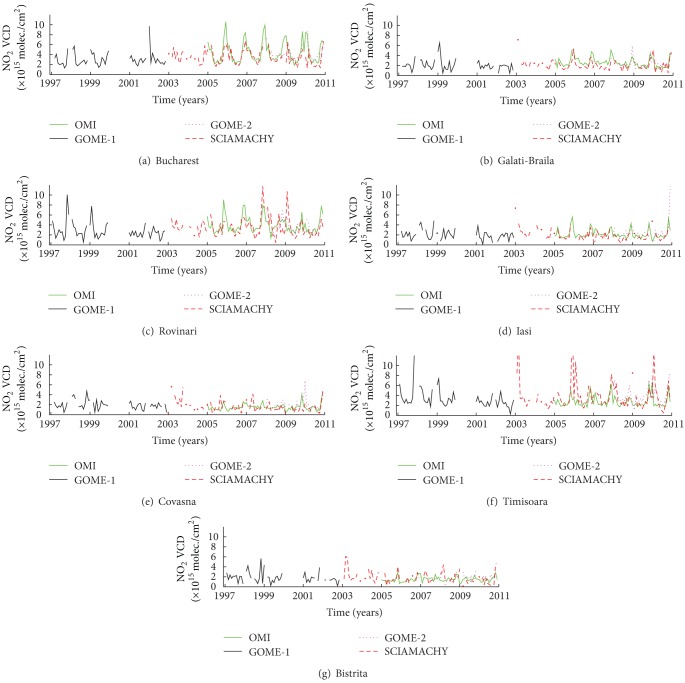
Time series of NO_2_ loading for all cities, for the entire period when satellite observation is available (1997–2010), using data from GOME, SCIAMACHY, OMI, and GOME-2.

Time series of monthly means of tropospheric NO_2_ columns derived from all satellite instruments for each city are shown in Figure 3. All satellite measurements indicate that the tropospheric NO_2_ column varies strongly with season, with minimum values during the summer and maximum values during the winter. Differences between seasons are due to both natural and anthropogenic causes. In [2], the lifetime of tropospheric NO_2_ is estimated to be 6 hours during the summer and 18–24 hours during the winter. The lifetime of NO_2_ increases when the temperature decreases and photolysis is slow. The anthropogenic contribution is higher during the winter due to heating and increased energy production.

Important gaps exist in GOME-1 measurements; thus, we will focus on SCIAMACHY, OMI, and GOME-2 data. All three satellites display the seasonal variation, which varies from point to point, function of pollution. The highest differences between cold and warm seasons are seen for cities with industrial activities and/or intense traffic, classified in this study as cities with high or medium pollution (Table 2).

Satellite results mostly agree, but there are also important differences especially for high loadings. An interesting observation is that OMI shows almost constantly higher values than the other two satellites during the winter only in Bucharest. Using in situ measurements, possible explanations will be presented in Section 4. The lowest loadings are observed in Bistrita and Covasna, while the highest loadings are seen in Rovinari and Bucharest. GOME-1 measurements are absent in 2000 everywhere. There is no clear trend in any of the time series. Some increase in NO_2_ loadings can be seen in Bucharest, which are probably caused by increased industrial activity and not by traffic, as we will show later. The main NO_2_ source in Rovinari is a power plant producing electricity and heating using fossil fuel. The production of electricity at Rovinari power plants is directly influenced by the demand of electricity, which reflects on NO_2_ emissions. The seasonal variation is influenced by the emission that resulted from the demand of electricity on the peak zone. Possible sources of NO_2_ in Galati-Braila and Iasi (Figures 3(d) and 3(e)) are an iron and steel factory, a power plant, and the traffic. The latter is relatively low (about one-third) in comparison with Bucharest. The number of vehicles in the cities of Galati-Braila is approximately 300.000 pieces while the vehicles number in Bucharest exceeds 1 million pieces, according to [22]. The seasonal variation exists but it is smaller (200%) than that in the previous two cities. Winter values are around 3-4 × 10^15^ molec./cm^2^, while summer values vary between 1 and 2 × 10^15^ molec./cm^2^. In these cases, the seasonal variation might be attributed mostly to NO_2_ lifetime, since no clear decreasing trend is observed in any of the time series, although activities from metallurgical industry from these cities have decreased year by year.

Covasna and Bistrita are small cities close to mountains but are considered to be rural because of missing industries and low traffic. The difference between the NO_2_ loadings in summer and winter is smallest because NO_2_ production in winter is due solely to fossil fuel (coal and wood) utilization at individual scale for heating buildings and houses. In comparison with other locations, these two cities present the smallest NO_2_ values and all instruments show similar values.

Figure 3 shows that the best data set is provided by the OMI instrument, with continuous measurements over a long time period (2005–2010); thus, OMI measurements should be used for assessing possible effects of point sources. This is due to a better resolution of the instrument (Table 1), continuous monitoring (no data gaps). Also, the retrieval algorithm is different. Data are obtained from GOME-2 and SCIAMACHY measurements using the retrieval algorithm calculated by KNMI/BIRA while OMI data sets are obtained using the DOMINO retrieval algorithm developed by KNMI [19, 20]. The differences between retrieval algorithms used in OMI and SCIAMACHY/GOME-2 are based on spectral fitting characteristics of NO_2_ retrievals. Since superimposed satellite data are not very informative, due to them being different in many cases, OMI measurements were selected for a more detailed analysis.

**Figure 4:**
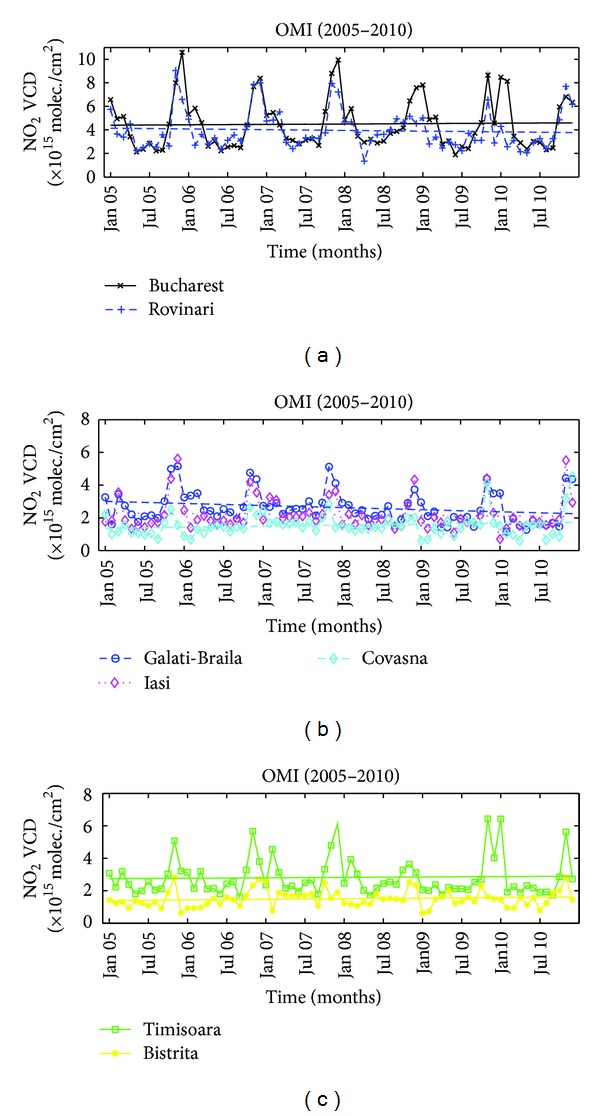
NO_2_ time series obtained with the OMI instrument over selected cities.

The monthly variation of the NO_2_ column measured with OMI over the seven selected cities is shown in Figure 4. Maximum values in winter reach 2.5 × 10^15^ molec./cm^2^ in clean areas and can go up to 10 × 10^15^ molec./cm^2^ in Bucharest. In summer, NO_2_ loading values are between 0.7 × 10^15^ molec./cm^2^ in clean areas and 2 × 10^15^ molec./cm^2^ in polluted regions. The seasonal variation of the NO_2_ column is clear at each site but is the highest at regions where the pollution level is high (500% in polluted areas versus 250% in clean areas). The lifetime of tropospheric NO_2_ varies by about 300% from summer to winter [23]; thus, one may assume that in clean areas only a small percentage of the seasonal change is due to increased fossil-based emissions.

Interestingly, the NO_2_ concentration is significantly lower in the winter of 2009 for all locations in the central and western part of Romania, regardless of the pollution level. This is not seen in the south and eastern part, that is, in Bucharest, Iasi, and Galati-Braila. This might suggest that the cause relates to some particular weather characteristics that affected the lifetime of NO_2_, possibly abnormally high temperatures on the western/central side.

There is no clear trend in the observed time series; the variation is relatively small and there is no indication of any clear correlation with the pollution level. The differences are relatively small and cannot be associated with anthropogenic or natural causes. Peaks in concentrations are reached in late autumn/early winter (November-December) at all locations while flat minima are seen during summer.

## 4. Discussions

**Figure 5:**
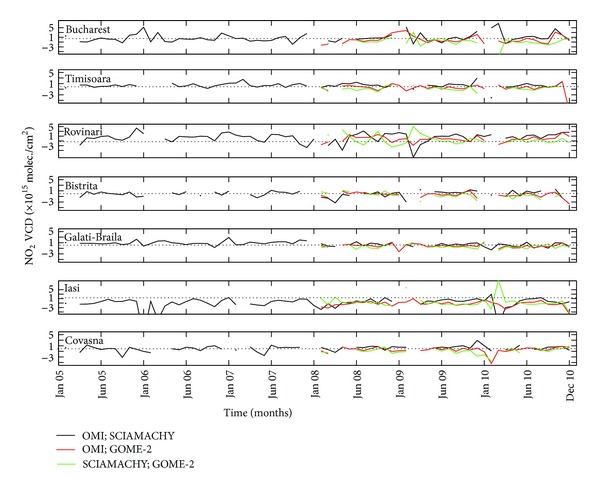
The differences between NO_2_ satellite measurements over all selected locations.

A relatively large scatter of satellite measurements is observed, especially during winter and/or for high concentrations. The difference between different satellite measurements is shown in Figure 5 for the period 2003–2011, in order to check whether the scatter varies with time, depending on the season or on the pollution level. GOME-1 data set is excluded because of the large data gaps and of the impossibility of achieving a comparison with other instruments. If the difference between satellite measurements is due to the different overpass time or to the horizontal resolution of each instrument, some repeatability should be observed, at least for high-level pollution area, where, generally, OMI should see higher values since the resolution is better. Large pixels in SCIAMACHY or GOME-2 data can introduce smoothing effects.

This is, indeed, the case for Bucharest and, partly, for Timisoara, Galati-Braila, and Rovinari. In the latter three cases, OMI measurements are also relatively high, but for a smaller percentage of time. Instances when the other two satellites show higher values are few. The differences between satellite measurements of the NO_2_ concentration in Bucharest can be larger than 2 × 10^15^ molec./cm^2^, especially in winter. One explanation could be that the OMI sensor passes over Bucharest around noon, when strong pollution episodes occur from industrial activities and/or traffic. The other two instruments see Bucharest 3 hours earlier, that is, in the morning. Thus, high values of NO_2_ loadings in SCIAMACHY or GOME-2 indicate that strong pollution events occurred during the previous night. This is seen sometimes in Rovinari, in 2008 and 2009, and in Iasi, Bistrita, and Covasna. Probably these results are connected with increased production of heating and electricity during winter in the evening/night. Since the lifetime of NO_2_ is longer in the winter, the two instruments could see higher NO_2_ loadings in the morning. However, an explanation for the differences between SCIAMACHY and GOME-2 is not straightforward, since both instruments have the same time of the pass, similar pixel size, and the same retrieval algorithm. In summer, due to the strong photochemical processes reducing the NO_2_ lifetime and to a decreased emission rate, values shown by the OMI sensor should be smaller than or equal to those given by the other two satellites. This is indeed seen during summers, especially in Bucharest.

**Figure 6:**
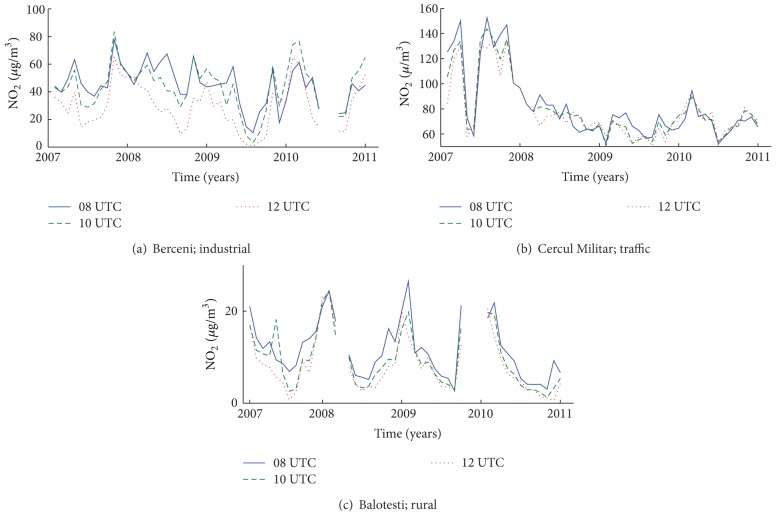
Daily variation of NO_2_ in three locations with different types of NO_2_ sources: industrial (power plant), urban (mainly traffic), and rural (no important source).

The main atmospheric pollution sources in Bucharest are heavy traffic and power plants using fossil fuel (~50%). Yearly, the variations of NO_2_ concentration measured in situ at three particular times of the day (8 UTC, 10 UTC, and 12 UTC) in Bucharest are shown in Figure 6 for several locations (industrial urban, rural, and traffic). Power plants in Berceni (industrial station, Figure 6(a)) create a strong accumulation of NO_2_ in winter, which has a peak in the morning. This coincides with the NO_2_ variation seen by OMI over Bucharest. Since the OMI sensor has the finest resolution pixel, a late overpass, and a different algorithm calculation, it is the closest to the in situ values for regions where strong NO_2_ point sources exist.

Results of traffic monitoring stations look different; the diurnal variation during the satellites overpass is small because the intense traffic is a constant NO_2_ source (Figure 6(b)) during 08–12 UTC. Since traffic pollution in Bucharest is high and the differences between the three curves are not important, we can assume that higher concentrations seen by OMI over Bucharest relate to industrial emissions. NO_2_ produced by traffic dropped significantly, from about 140 *μ*g/m^3^ to about 70–80 *μ*g/m^3^, in 2008. NO_2_ maxima are generally observed in spring and autumn while during winter and summer the traffic seems to be reduced. This is another indication of the fact that none of the three instruments correctly reproduces NO_2_ originating from traffic.

**Figure 7:**
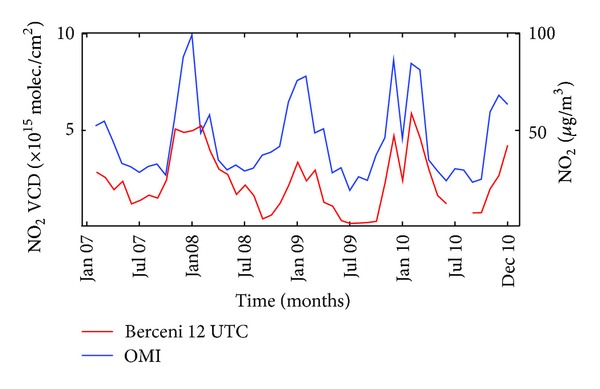
Comparison between OMI observations and in situ measurements for the Berceni (industrial) area in Bucharest at 12 UTC.

Correlation coefficients between OMI data and in situ data at the three stations, at three moments of the day, were calculated and are shown in Table 3. Obviously, NO_2_ caused by traffic is not correctly evaluated by OMI. Interestingly, the best correlation (0.76) exists for the in situ measurements at 12 UT, which is exactly the time when OMI passes over Bucharest. This is also seen in Figure 7, where OMI and in situ measurements are plotted. There is an almost hand-in-hand variation of the two time series; all departures from the seasonal variation shown by in situ measurements are mirrored in OMI results.

**Figure 8:**
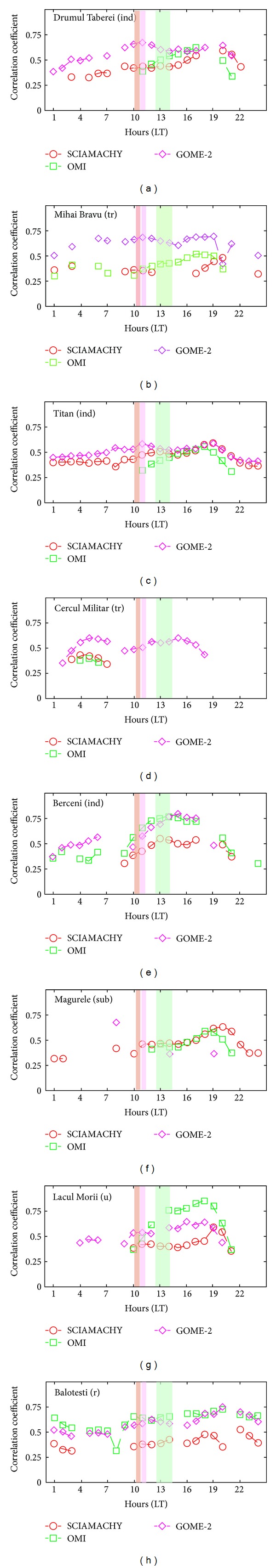
Diurnal variation of significant correlation (*P* < 0.05) between local, in situ, measurements and corresponding satellite records. Coloured rectangles point to the local time when the satellites pass over Bucharest: SCIAMACHY (light red) and GOME-2 at 11 LT (light magenta) and OMI at 13 LT (light green).

In order to see whether satellite-based measurements of NO_2_ concentration might depend on the time of satellite pass, correlation coefficients between NO_2_ concentrations measured at each of the eight monitoring stations on an hourly basis, on one hand, and the three available satellite estimations, on the other hand, are plotted as a function of the local time in Figure 8. The stations are classified as follows: traffic monitoring (tr), industrial (ind), urban (u), suburban (s), and rural (r). Only significant correlations (*P* < 0.05) are shown. Some of the correlations vary with the local time, but the maxima of correlations are not always located at the time of satellite pass, as one would expect.

Figure 8 shows that satellite measurements are good indicators for rural/urban areas, which have a reduced pollution level. Also, the satellite instruments could be used for the evaluation of industrial emissions. Opposingly, NO_2_ originating from traffic is not well depicted by satellites. The correlation between in situ and satellite measurements seems to be the best for GOME-2 over most of the stations. OMI measurements (green line) correlate well with in situ measurements in urban and rural areas but also for industrial areas (Berceni), while GOME-2 provides good estimates for traffic and industrial activities. A part of the correlation is certainly due to the seasonal effect, but this should not contribute too much since in situ variations (see Figure 6) are much noisier than satellite data, especially for industrial locations.

## 5. Conclusions 

In this paper, we have analyzed the temporal and spatial characteristics of the NO_2_ concentration measured by satellite instruments over Romania, at seven different locations, which are representative for various pollution levels. The maximum monthly mean concentration of tropospheric NO_2_ over Romania between 1997 and 2010 was about 11 × 10^15^ molec./cm^2^ and was recorded by OMI over Bucharest city in December 2006. The minimum monthly value, less than 1 × 10^15^ molec./cm^2^, was recorded by OMI over the mountain regions where Bistrita and Covasna cities are located.

We show that measurements with satellite instruments can provide valid information about the variation or trend in NO_2_ atmospheric pollution over different urban areas with different levels of atmospheric pollution. Seasonal variation is higher for polluted areas but is clear for all locations. Differences between satellite measurements are sometimes important and relate, most likely, not only to the type of NO_2_ source (industrial, traffic, and urban activities) but also to the difference in pixel size and in the time of the pass. Careful analysis of these differences might help in identifying the most probable source of NO_2_ at tropospheric level at a particular location, taking into account the NO_2_ lifetime, which is strongly connected with the environmental temperature. NO_2_ loadings in OMI measurements are generally higher than those provided by SCIAMACHY and GOME-2 especially over highly polluted areas, where the NO_2_ source has a diurnal variation. Opposingly, the latter measurements indicate higher values of the NO_2_ concentration above locations with low industrial activity where the main NO_2_ source is housing heating using fossil fuels.

Correlation analysis suggests that the OMI sensor can reasonably detect NO_2_ variation in industrial areas (i.e., caused by big power plants or other factories (steel factories)). Due to its pixel (13 × 24 km^2^) and time pass, the OMI sensor does not evaluate correctly the NO_2_ pollution caused by traffic. All satellites seem to be good indicators for the NO_2_ level in rural, nonpolluted areas.

There is no clear NO_2_ trend in any of the seven time series. Important increases or decreases in the NO_2_ concentration do exist and are, most likely, linked to local natural and anthropogenic particular conditions.

## Figures and Tables

**Table 1:** Instruments aboard satellites used in the analysis.

Instrument	Start date	End date	Horizontal resolution	Global coverage	Bucharest overpassing time/UTC

GOME-1	04/1996	06/2003	320 × 40 km^2^	3 Days	08:45–09:45
SCIAMACHY	07/2002	12/2010	60 × 30 km^2^	6 Days	08:20–09:20
OMI	10/2004	12/2010	13 × 24 km^2^	1 Day	10:15–12:30
GOME-2	04/2007	12/2010	80 × 40 km^2^	1 Day	08:55–09:45

**Table 2:** Coordinates and pollution level for selected cities.

City	Latitude (°N)	Longitude (°E)	Pollution level
Bucharest	44.436	26.127	High
Rovinari	44.912	23.162	High
Timisoara	45.749	21.227	Med
Iasi	47.162	27.588	Med
Galati-Braila	45.439	28.003	Med
Covasna	45.850	26.183	Low
Bistrita	47.133	24.483	Low

**Table 3:** Correlation coefficients (top) between OMI and in situ measurements of the NO_2_ observations and their significance (bottom) for three locations: rural, industrial, and traffic.

Correlation coefficients between OMI and in situ measurements		
Balotesti 8 UTC-OMI *R* = 0.6422 *P* = 0.0001	Balotesti 10 UTC-OMI *R* = 0.6171 *P* = 0.0001	Balotesti 12 UTC-OMI *R* = 0.6512 *P* = 0.0001

Berceni 8 UTC-OMI *R* = 0.5641 *P* = 0.0001	Berceni 10 UTC-OMI *R* = 0.7257 *P* = 0.0001	Berceni 12 UTC-OMI *R* = 0.7644 *P* = 0.0001

Cercul 8 UTC-OMI *R* = 0.0342 *P* = 0.8173	Cercul 10 UTC-OMI *R* = 0.0797 *P* = 0.5900	Cercul 12 UTC-OMI *R* = 0.1581 *P* = 0.2833
